# Upper Gastrointestinal Bleeding Caused by Rupture of Pancreatic Pseudoaneurysms

**DOI:** 10.1155/2023/7888990

**Published:** 2023-09-27

**Authors:** Huong Tu Lam, Thang Dinh Nguyen, Phat Tan Ho, Ai Van Nguyen, Tuan Huynh Nhat Nguyen, Phuoc Van Le, Thong Duy Vo

**Affiliations:** ^1^Department of Internal Medicine, Faculty of Medicine, University of Medicine and Pharmacy at Ho Chi Minh City, Ho Chi Minh, Vietnam; ^2^Department of Gastroenterology, Cho Ray Hospital, Ho Chi Minh, Vietnam; ^3^Department of Radiology, Cho Ray Hospital, Ho Chi Minh, Vietnam

## Abstract

Pseudoaneurysm rupture in patients with pancreatitis is a rare but fatal etiology of upper gastrointestinal bleeding. We report a rare case of upper gastrointestinal bleeding in a patient who presented simultaneously with two pseudoaneurysms, a potential cause of severe gastrointestinal bleeding. Angiography was successfully performed with coil embolization of the target arteries and both pseudoaneurysmal sacs. The patient was discharged 9 days after admission without further events within a 3-month follow-up period.

## 1. Introduction

Pseudoaneurysmal bleeding is a rare but fatal complication of pancreatitis. Once pancreatic pseudoaneurysms rupture, the mortality rate can exceed 40%. Meanwhile, the clinical presentation can range from abdominal pain (due to the rapid enlargement of a pseudocyst), overt bleeding, or self-limiting episodes of bleeding to rapidly progressing and life-threatening hemorrhage that induces a difficult, delayed diagnosis and management. Here, we present the case of a patient who experienced several episodes of hematemesis due to pancreatic pseudoaneurysm rupture.

## 2. Case Presentation

A 34-year-old male with a history of alcohol abuse presented to our hospital with a 2-day history of severe, constant epigastric pain that radiated to the back, nausea, and two episodes of hematemesis (approximately 400 mL of bright red blood in total). He was transfused with two units of packed red blood cells at a district hospital and referred to our hospital.

He drank alcohol daily for 12 years (24 g of alcohol per day) and reported several episodes of epigastric pain that responded to self-treatment with antacids. He had no history of pancreatitis, melena, or hematemesis. Physical examination showed tachycardia (136/min), blood pressure of 100/60 mmHg, respiratory rate of 20/min, a temperature of 37°C, regular rhythm, clear lungs, and epigastric tenderness on palpation with no peritonitis signs.

**Figure 1:**
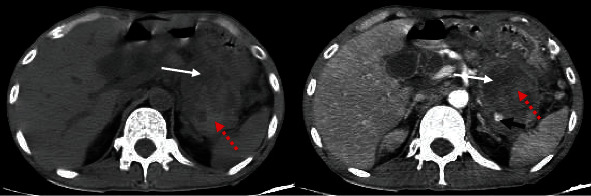
Axial contrast-enhanced computed tomographic images reveal a pseudoaneurysm at the pancreatic tail-omental bursa region adjacent to the gastric wall, which might be the cause of the bleeding (white arrow: pancreatic pseudocyst; black arrow: pseudoaneurysm lumen filling with contrast; red dashed arrow: thrombus).

**Figure 2:**
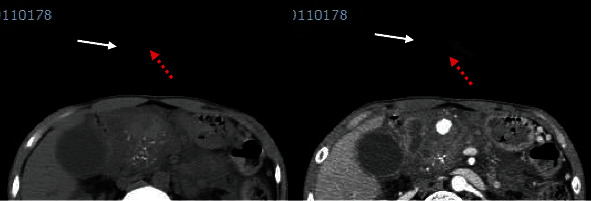
Axial contrast-enhanced computed tomographic images demonstrate a second pseudoaneurysm arising from the gastroduodenal artery, suggesting recent and ongoing bleeding (white arrow: pancreatic pseudocyst; black arrow: pseudoaneurysm filling with contrast; red dashed arrow: thrombus).

**Figure 3:**
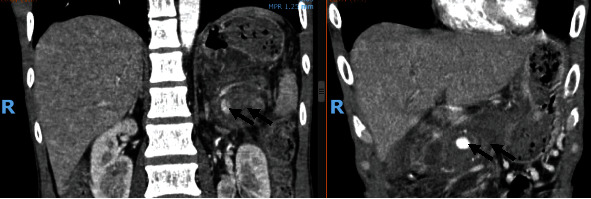
Coronal contrast-enhanced computed tomographic images reveal two pseudoaneurysms (black arrows: pseudoaneurysm filling with contrast).

An urgent endoscopic evaluation followed by initial resuscitation was performed at admission due to repeated rebleeding episodes. Esophagogastroduodenoscopy (EGD) revealed a small Forrest III duodenal ulcer and a small amount of dark red blood in the corpus. However, this small lesion was inconsistent with the patient's clinical picture when four units of blood were transfused. A repeat upper endoscopy was performed by an experienced endoscopist to identify lesions potentially overlooked at the time of initial endoscopic evaluation. The second endoscopy also revealed a small, shallow duodenal ulcer and a large amount of blackish-red blood in the stomach; therefore, computed tomography (CT) angiography was promptly performed as the next step (Figures 1–3). Simultaneously using lipase testing and abdominal ultrasound, we investigated the cause of gastrointestinal bleeding via endoscopy and observed the etiology of acute epigastric pain in a patient with a history of alcohol abuse. Laboratory findings are summarized in Table 1. Abdominal ultrasonography revealed pancreatic duct dilation and various shadowing pancreatic ductal stones, suggesting chronic pancreatitis.

**Figure 4:**
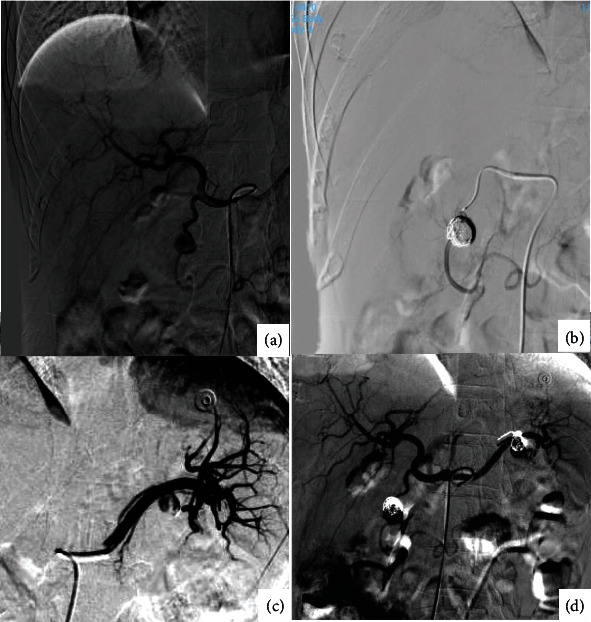
Assessing the vascular supply of the pseudoaneurysms by angiography followed by coil embolization. (a) A pseudoaneurysm arising from the gastroduodenal artery. (b) Coil embolization of the target artery and the pseudoaneurysmal sac. (c) A pseudoaneurysm arising from the short gastric artery. (d) Successful coil embolization of the target arteries and both pseudoaneurysm sacs.

CT angiography revealed two pseudoaneurysms, which could cause bleeding. After consulting with the interventional radiology and gastroenterology teams, the patient was referred to the interventional radiology department for emergency angiography with intervention (see Figure 4).

Digital subtraction angiography (DSA) revealed two pseudoaneurysms (12 mm × 12 mm and 12 mm × 11 mm) arising from the gastroduodenal and short gastric arteries, respectively. Five coils were deployed to occlude the target arteries and the pseudoaneurysmal sacs. Finally, the patient was successfully treated endovascularly using coil embolization.

After the intervention day, gastrointestinal bleeding was controlled, with no evidence of hemorrhage. The patient did not present with new bleeding episodes or other complications from the procedure within the 3-month follow-up period.

## 3. Discussion

Upper gastrointestinal bleeding in patients with pancreatitis has various causes. Cases unrelated to pancreatitis include peptic ulcers, Mallory–Weiss tears, esophagitis, and varices from concomitant alcoholic cirrhosis. Other etiologies directly associated with pancreatitis include bleeding from a pancreatic pseudocyst, a pseudoaneurysm, and portal or splenic vein thrombosis. The pseudoaneurysm can rupture into an associated pseudocyst (thereby converting the pseudocyst into a larger pseudoaneurysm) or directly into the adjacent hollow organs, peritoneal cavity, or pancreatic duct [1]. Many visceral arteries may be involved, with the splenic artery being the most common, followed by gastroduodenal or pancreaticoduodenal, gastric, and hepatic arteries (in that order) [1, 2].

The clinical presentation can vary from abdominal pain (due to the large pseudocyst) and unexplained anemia to several forms of bleeding, such as overt bleeding (when rupturing into the gut through the pancreatic duct), self-limiting episodes, or massive hemorrhage [1]. Once unexplained gastrointestinal bleeding occurs in a patient with pancreatitis or a known pseudocyst, a diagnosis of pseudoaneurysm should be immediately considered, and urgent upper endoscopy should be performed. However, bleeding from the ampulla of Vater is rarely observed, and the next step is evaluation using CT with intravenous contrast [1, 3]. When a pseudoaneurysm is identified, it should be treated whether or not it has caused bleeding. In this situation, DSA is performed to localize bleeding pseudoaneurysms and perform angioembolization, and surgery is indicated after the failure of endovascular techniques [1]. In a study of 980 patients with acute or chronic pancreatitis from 2010 to 2016, Mallick et al. reported that 46 (4.7%) patients developed pseudoaneurysms; the most common clinical presentation was gastrointestinal or intra-abdominal bleeding in 37 (80.4%) patients, and most cases were treated with nonsurgical management, including embolization in 31 (67.4%) and percutaneous thrombin injection in nine (19.6%) patients [4].

Our patient, with a history of alcohol abuse, presented to the emergency department with several episodes of hematemesis; however, the lesions observed on endoscopy were inconsistent with the symptoms. In addition, his abdominal pain was likely due to pancreatitis; therefore, CT angiography was indicated, and the result revealed two pancreatic pseudoaneurysms. It takes at least 5 years (and, in most patients, >10 years) of alcohol consumption exceeding 4–5 drinks per day to develop chronic pancreatitis [1]. Our patient had drunk for 12 years (24 g of alcohol per day) and also had some recurrent attacks of the same abdominal pain; the abdominal ultrasound of this case suggested chronic pancreatitis, so these pseudoaneurysms may be more likely to be associated with an acute than chronic pancreatitis in an alcoholic patient. Angiography was promptly performed, with coil embolization of the target arteries and both pseudoaneurysmal sacs.

In patients with pancreatic pseudoaneurysm complications, mortality may exceed 40% once bleeding occurs, depending on the severity of blood loss and other coexisting conditions [1]. If the patient received only basic supportive treatment, the mortality rate is up to 90%. Even after pseudoaneurysm surgery, the mortality rates vary between 20% and 30%. Mortality rates were highest when the pseudoaneurysm was located in the head of the pancreas. Interventional embolization increases the success rate, but the rebleeding rate remains high, and the overall mortality rate is approximately 16% [5]. This patient was discharged without further events within a 3-month follow-up period.

## 4. Conclusion

Our case report adds to pancreatic pseudoaneurysm complications; mortality may exceed 40% once bleeding occurs, depending on the severity of blood loss and other coexisting conditions. However, this is the first case demonstrating that successful interventional embolization increases the success rate. In addition, this case also demonstrates complete upper gastrointestinal bleeding caused by rupture of pancreatic pseudoaneurysm recovery.

## Figures and Tables

**Table 1:** Laboratory characteristics of the patient.

Laboratory tests	Result	Reference ranges
Hemoglobin (g/L)	66	120–170
Hematocrit (%)	20.5	34–50
White blood cell (g/L)	9.08	4–11
Platelets (g/L)	327	200–400
Lipase (U/L)	144.4	<78
Blood urea nitrogen (mg/dL)	11	7–20
Creatinine (mg/dL)	0.76	0.7–1.5
Aspartate aminotransferase (U/L)	104	9–48
Alanine aminotransferase (U/L)	42	5–49
Total bilirubin (mg/dL)	3.07	0.2–1
Direct bilirubin (mg/dL)	1.48	0–0.2
Albumin (g/L)	25.0	35–45
Gamma-glutamyl transferase (U/L)	407	4–38
Alkaline phosphatase (U/L)	517	46–116
Triglyceride (mg/dL)	54	35–160

## Data Availability

There is no data availability to be reached. All of the information is supplied in the manuscript.
